# How Can the Implementation of Ethical Norms Be Guaranteed in Biomedical Studies?

**Published:** 2020

**Authors:** Mohammad Reza Sadeghi

The development of medical sciences is due to efforts of scientists and clinicians to conduct various studies to answer questions and solve the health problems. Currently, scientists in organizations, institutes, and universities are trying for recognizing priorities in scientific discovery and improving medical sciences through increasing their scientific output, as well as raising their national and international rankings. Therefore, the role of scientific studies and subsequently published articles in the progress and development of countries is obvious to everyone. But the history of scientific studies during the last century shows that human standards, human rights and ethical considerations have not been met in a large number of human researches particularly in the field of medicine and biosciences. Also, it sometimes led to irreparable damage to the scientific body of society and even to the scientific position of academic staff. Therefore, ethical obligation of submitted manuscript is a critical criterion for acceptance and publication of biomedical findings. In addition to numerous daily errors in medical practice worldwide, a large number of unsuccessful and useless clinical studies were the main reason for the development of global rules, documents and guidelines which evolved into ethical norms and code of practice.

The ethics in research was established in anthropology field at first to support and protect the rights of cases under investigation, as well as to protect researchers from unreliable and unsafe events that jeopardize their comfort. The first major effort for protection of human rights was made in June 1964 as The Declaration of Helsinki. It was followed by seven subsequent revisions (The most recent one in October 2013); consequently, 11 paragraphs in the original version reached to 37 paragraphs in the 2013 version. It is the most important document of ethics in research, which forms the basis for many subsequent documents (1).

Most of scientific journals follow the standard ethical guidelines such as the ones established by the International Committee of Medical Journal Editors (ICMJE) for standardizing the ethics, preparation and formatting of manuscripts submitted for publication by biomedical journals. They banned acceptance and publication of articles which neglect ethical issues such as informed consent, ethical committee approval, confidentiality of study participants, conflict of interest, double publications, plagiarism, uncertainty in the authorship, unethical research behaviors, data falsification, deliberate misbehavior in data presentation that may lead to harms for community and ultimately the trust to scientific society (2).

Publication of an article is the final phase of a scientific project that has been performed for a long period following various evaluations and consuming a lot of budget and resource which is borne to society. Therefore, scientists are expected to publish the results of their work in complete honesty and trust. However, a large number of submitted manuscripts violate ethical standards and most of them are rapidly rejected by journal’s editorial board. Ethical behavior in biomedical journals is the greatest responsibility of the authors, in the first place, and the reviewers, editor and publishers, in the second, although it should be understood that unintentional errors are inevitable and must be distinguished from deliberate practice. In addition, the ethical committees are also responsible for supervision of their own approved researches and creating a healthy environment for conducting ethics norms (3).

Most of prepared manuscripts are free of ethical misconducts and errors. However, despite careful supervision of ethical committees and peer review process, ethical norms have been compromised in some published articles, which have been identified over the time through reanalysis of articles by the audit trails or editorial board. Few of them may be corrected through formats such as “Expression of Concern”, “Erratum” Corrigendum or, the cases with deliberate gross error are retracted. If it was allowed to publish the results of studies lacking ethical norms, it could increase the motivation of some researchers to circumvent and disregard ethical norms in their research, or at least it could act as a deterrent to prevent unethical research. On the other hand, the current trend of some journals to publish editorials and criticism for not adhering to ethical codes in research seems to be ineffective, since with the online publication of the most journals, most of people search their articles in the databases and are less likely to look for editorial, comments or letters to editor (4).

So raised question is that whether the method of blaming researchers who violate ethical norms in their study is an optimal deterrent to prevent the occurrence of such research in the future, especially in deliberate cases. Surely, the answer to this question is negative, as there are numerous editorial comments, amendments, erratum, and letters to the editor on the necessity of ethics in biomedical research. In spite of this, we are faced with a great deal of manuscripts that due to ethical misconducts are quickly rejected or quite reverse, accepted with great ignorance and even may be hidden from the view of referees and editors and subsequently they are published. So again, the point at issue is about the useful strategies which the journals may put into practice in dealing with such articles to have a greater preventive effect.

Perhaps, one of the suggested solutions is to publish such manuscripts submitted for publication in academic journals, but not like the usual papers. Rather, designing and creating a specific database seems to be an optimal strategy. Such database can be created by the World Association of Medical Editors (WAME), ICMJE, Cope, PubMed, or any other international organization in the field of publishing scientific researches, for publishing articles with major ethical issues. The database will also require an expert team and reviewers to evaluate and verify the allegations and provide views and commentary on documents for audience education. On the other hand, the number of papers of a researcher submitted to the database can be recorded as a negative index for their academic status. Currently, indicators such as H.Index, G.Index, Cumulative IF, etc. have their place as a positive index that researchers are trying to promote them. In contrast, academic staff will try to be bound to ethical norms in their researches in order not to harm their scientific position.

The publication of such articles, as well as the deterrent role for researchers and clinicians will provide useful practical training on how to apply ethical norms in future studies for other researchers. In addition, universities, institutes and ethics committees will be more focused and motivated on the evaluation, approval and monitoring of referred proposals. Ultimately, it will prevent waste of resources for conducting useless and futile research and also the potential harm to individuals due to false results of such studies.
